# Modulation of Stem Cell Survival and Engraftment: Implications for Stem Cell-Based Therapy

**DOI:** 10.7150/thno.120805

**Published:** 2025-08-11

**Authors:** Qingwen Xu, Chaochao Zhang, Xinyu Liu, Yuyao Sun, Bowei Li, Sihui Nian, Yan Wang, Chenggong Yu

**Affiliations:** 1 Department of Pharmacy, the First Affiliated Hospital of Wannan Medical College (Yijishan Hospital of Wannan Medical College), Wuhu 241001, China.; 2 School of Pharmacy, Wannan Medical College, Wuhu 241002, China.; 3 Organoid Innovation Center, CAS Key Laboratory of Nano-Bio Interface, Division of Nanobiomedicine, Suzhou Institute of Nano-Tech and Nano-bionics, Chinese Academy of Sciences, Suzhou 215123, China.; 4 Center for Xin'an Medicine and Modernization of Traditional Chinese Medicine of IHM, Wannan Medical College, Wuhu 241002, China.; 5 State Key Laboratory of Neurology and Oncology Drug Development, Nanjing, China.; 6 Anhui Innovative Center for Drug Basic Research of Metabolic Diseases, Wannan Medical College, Wuhu 241002, China.

**Keywords:** stem cell therapy, metabolic remodeling, glucose delivery, oxidative stress injury, stem cell spheroids

## Abstract

Stem cell transplantation holds promise for the treatment of degenerative diseases, tissue injuries, and malignancies. Despite this potential, clinical outcomes have often fallen short, largely due to limited survival and engraftment of transplanted cells at target sites. Recent research efforts have focused on optimizing cell-based therapies through improved understanding of stem cell biology and responsiveness to environmental cues. Emerging evidence indicates that the hostile post-transplantation microenvironment contributes to irreversible cellular damage and death, driven by metabolic dysfunction, immune-mediated responses, reactive oxygen species (ROS), altered biomechanical rigidity, and disrupted intercellular communication. To address these challenges, various strategies have been explored, including supplementation with exogenous metabolic substrates, enhancement of vascular remodeling, administration of antioxidants, and the application of three-dimensional (3D) stem cell spheroids. This review synthesizes current approaches aimed at improving cell viability and therapeutic efficacy in regenerative medicine.

## Introduction

Stem cell-based therapy has emerged as a groundbreaking strategy for the treatment of malignancies, neurodegenerative diseases, organ fibrosis, and tissue injuries 1, 2. Unlike conventional small-molecule drugs, which typically act through a single molecular pathway, stem cells (SCs) mediate therapeutic effects *via* multilineage differentiation and paracrine signaling mechanisms 3, 4. Although numerous preclinical and clinical studies support the potential of SCs as a novel therapeutic modality, clinical translation remains significantly limited by poor post-transplantation cell survival and engraftment 5-7. Studies indicate that up to 90% of transplanted SCs undergo apoptosis within the initial days post-transplantation, primarily due to the hostile microenvironment and disruption of cellular homeostasis 8-10. This substantial cell loss compromises therapeutic efficacy, reproducibility, and stability—factors critical to the quality standards expected of any therapeutic intervention.

Transplanted SCs encounter a multitude of environmental stressors that collectively contribute to early cell death. Among the most acute is ischemia-reperfusion injury, stemming from inadequate vascular supply at the transplantation site 11. In contrast to resident cells supported by an intact vascular network, transplanted SCs initially lack vascular connections and must survive until neovascularization occurs or integration into host vasculature is achieved 11, 12. This interim phase induces a metabolic crisis characterized by severe hypoxia, nutrient deprivation, and the accumulation of cytotoxic waste products 13, 14. To mitigate this, strategies such as direct oxygen and nutrient supplementation, as well as metabolic preconditioning *via* starvation-mimicking interventions, have shown promise in preserving short-term cell viability 15. Long-term survival, however, requires robust vascular reconstruction—either through cytokine-mediated angiogenesis or the deployment of biomaterial scaffolds designed to support vascular network formation 16-18. Oxidative stress represents another major obstacle to SC survival, driven by excessive ROS that exceed the cell's intrinsic antioxidant capacity 19. The abrupt transition from optimized *in vitro* conditions to the pathological oxidative environment of damaged tissues renders transplanted SCs particularly susceptible to redox imbalance 20. Countermeasures have included both genetic modifications to enhance endogenous antioxidant defenses and the delivery of ROS-scavenging components 21, 22. Furthermore, recent advancements in three-dimensional (3D) culture techniques for SCs and hydrogel scaffolds have emerged as effective strategies to better replicate the *in vivo* microenvironment, thereby addressing limitations inherent to traditional two-dimensional (2D) cultures. The 3D architecture of SCs—such as spheroids, aggregates, and biomimetic microstructures constructed through biological 3D-printing—preserves their multidirectional differentiation potential and enhances both cell-cell and cell-matrix signaling. These features facilitate intercellular interactions and result in cellular characteristics that more closely resemble those observed *in vivo* 23, 24. In this field, hydrogel scaffolds with defined physicochemical and biological properties have become a focal point in stem cell-based regenerative medicine. By combining structural support with the incorporation of biological factors 25, these scaffolds enable the construction of large-scale tissue constructs, offering potential for the artificial fabrication of complex organs 26-28.

**Figure 1:**
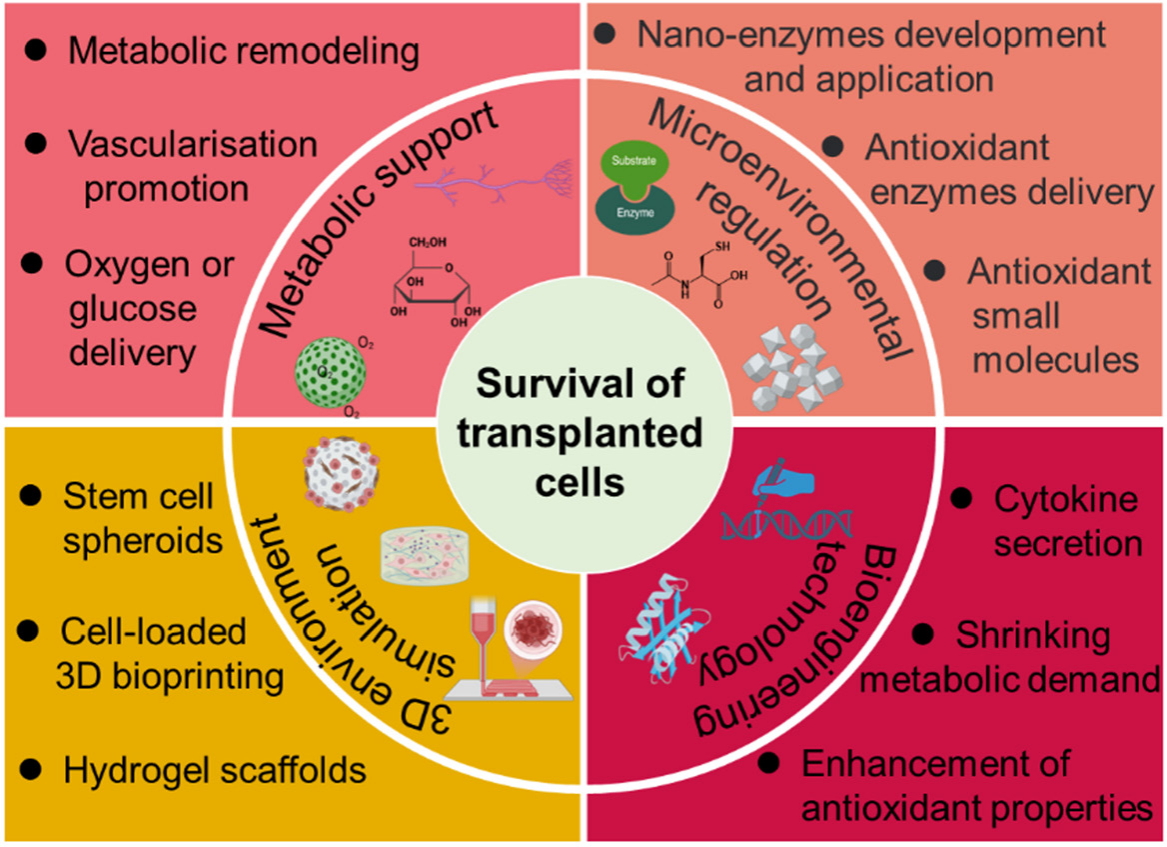
Overview of established strategies and emerging research approaches aimed at enhancing the survival and functional integration of transplanted SCs.

Over the years, various methods have been developed to address the challenges contributing to SC death. This review provides a comprehensive examination of current strategies aimed at improving stem cell survival post-transplantation (**Figure 1**), with particular focus on metabolic support, angiogenesis promotion, oxidative stress mitigation, and microenvironmental engineering. Deeper insight into the determinants of SC viability and integration may uncover novel therapeutic targets beyond those currently explored. Through continued interdisciplinary collaboration and innovation, it may be possible to overcome existing barriers and fully harness the regenerative potential of stem cell-based therapies for a wide array of debilitating conditions.

## Factors and Strategies for Modulating the Survival of Transplanted Cells

### Metabolism of SCs

Cellular metabolism serves as the biochemical foundation sustaining all life activities by generating energy through substrate utilization, synthesizing structural components, and eliminating metabolic waste to maintain homeostasis 29, 30. Unlike resident cells, transplanted SCs face significant metabolic constraints due to the absence of vascular support at the graft site 31. This vascular deficiency leads not only to an insufficient supply of essential substrates—including oxygen, glucose, fatty acids, and amino acids—critical for energy production *via* glycolysis and oxidative phosphorylation 32, 33, but also hampers the clearance of metabolic waste products. The resulting disruption in intracellular homeostasis promotes cellular stress and death. These interconnected metabolic limitations form a critical barrier to the survival of transplanted SCs.

#### Stem cell domestication

Abrupt changes in the microenvironment at transplantation sites significantly disrupt SC metabolism, ultimately causing cell death. According to previous research, preconditioning SCs to adapt to such adverse conditions enhances both survival and therapeutic efficacy. Extensive studies have demonstrated that hypoxic preconditioning (1-5% O₂) activates hypoxia-inducible factor (HIF-1α), which in turn upregulates pro-survival genes (*e.g.*, *VEGF*, *GLUT-1)* and antioxidant enzymes such as SOD2, thereby strengthening the anti-apoptotic capacity of SCs 34-39. For example, Beegle *et al.* found that mesenchymal stem cells (MSCs) preconditioned at 1% O₂ for 48 hours showed twice the survival rate under serum-deprived conditions compared to normoxic (20% O₂) controls 36. Similarly, Wan *et al*. demonstrated that hypoxia preconditioning significantly enhances vascular endothelial growth factor (VEGF) secretion by adipose-derived stem cells (ADSCs), improving the viability, proliferation, migration, angiogenesis, and glycolysis of human umbilical vein endothelial cells (ECs) (HUVECs), ultimately promoting urethral reconstruction through the upregulation of angiogenic and glycolytic pathways 40. This enhancement in resilience is attributed to hypoxia-induced metabolic reprogramming, characterized by a shift from oxidative phosphorylation to glycolysis, accompanied by increased glucose uptake, elevated lactate production, and reduced oxygen consumption 34. In addition, transient serum deprivation induces autophagy, which facilitates the removal of damaged proteins and organelles while upregulating protective heat shock proteins (*e.g.*, HSP70) to reduce stress-induced injury 41. Moya *et al.* further demonstrated that reducing metabolic demand prior to transplantation enables MSCs to tolerate extreme hypoxia (2% pO₂ for 75 days) and near-anoxia (0.1% pO₂ for 14 days) 42, 43. Importantly, this strategy preserves the proliferative capacity, differentiation potential, and paracrine function of SCs, rendering it a clinically feasible and cost-effective strategy for cell-based therapies.

#### Oxygen supplement

Oxygen tension has a direct effect on cellular energy metabolism, and consequently, affects both cell survival and therapeutic efficacy. Previous studies have shown that oxygen tension in rodent hearts decreases from 2.6%-3.3% to 0.9% pO_2_ within the first 5 minutes following arterial occlusion in rodent hearts, reaching near-anoxic conditions (0.2% pO_2_) after 30 minutes of ischemia 44, inevitably resulting in cellular necrosis within the ischemic tissues. To enhance SC survival and function at transplantation sites, hyperbaric oxygen therapy (HBOT) was initially employed to elevate tissue oxygen partial pressure 45. However, this approach lacks tissue specificity, and the resulting excessive oxygen levels may induce ROS-mediated damage 46. Hemoglobin-based oxygen carriers (HBOCs) have demonstrated potential in boosting MSC proliferation by 25% 47, yet their clinical utility remains limited due to ROS generation during oxygen release 48. To overcome these drawbacks, Cook *et al*. developed a novel oxygen delivery strategy involving hyperbaric oxygen loading and subsequent controlled gas release into the surrounding environment to support cell viability 49. Nevertheless, this method exhibits limited oxygen-loading capacity and permits only short-term oxygen release.

**Figure 2:**
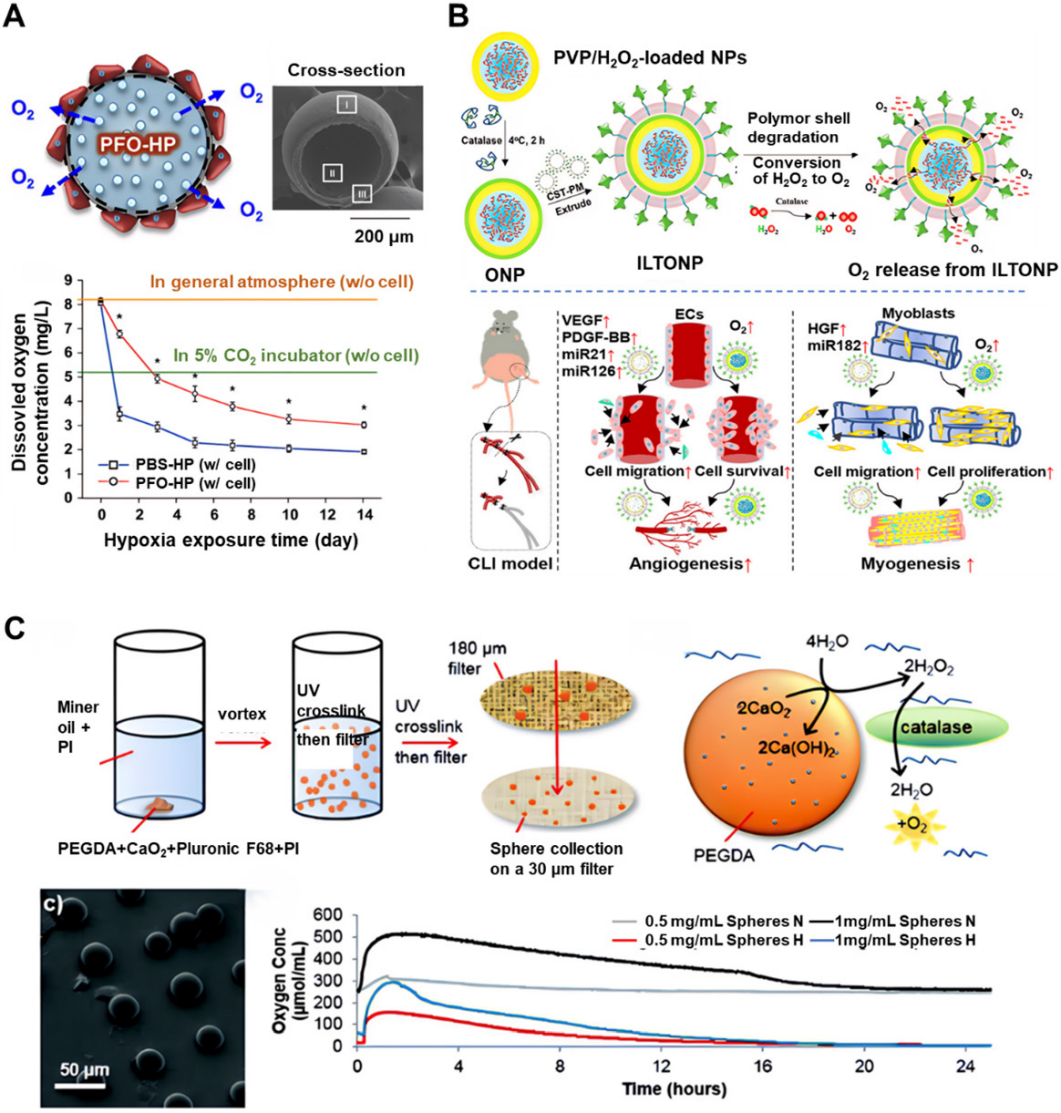
Representative examples of oxygen delivery and release system developed to support SC survival. (A) Schematic representation of perfluorooctane emulsion (PFO)-loaded hollow microparticles (HPs) designed for oxygen delivery under hypoxic conditions. The accompanying graph illustrates the concentration of dissolved oxygen in the cell culture medium surrounding the HPs during incubation in a hypoxic environment. Adapted with permission from 54, Copyright 2015 Elsevier B.V. (B) Schematic representation of the fabrication process and mechanism of action of ischemic limb-targeting oxygen-releasing nanoparticles (ILTONP) for the treatment of critical limb ischemia. Adapted with permission from 15, Copyright 2023 American Chemical Society. (C) Schematic depiction of PEGDA/CaO₂ microspheres and their corresponding oxygen release profiles at various concentrations under normoxic (N) and hypoxic (H) conditions. Adapted with permission from 59, Copyright 2018 The Royal Society of Chemistry.

With advancements in materials science, perfluorocarbons (PFCs) have been identified as promising oxygen carriers due to their oxygen solubility being 15-20 times greater than that of water 50. Their compatibility with microspheres and hydrogels presents opportunities for enhancing oxygen-carrying capacity and prolonging oxygen release duration 51, 52. For instance, PFC-hydrogel systems have been shown to enhance osteoblast viability under hypoxic conditions 53, while PFC-laden scaffolds have increased bone formation by 2.5-fold in defect models (**Figure 2A**) 54. However, the clinical application of low-molecular-weight PFCs is hindered by their rapid clearance and short *in vivo* retention time. This has prompted the development of modified PFCs through conjugation with hydrogels 55 or graphene oxide 56. Niu *et al*. designed fast-gelling hydrogels synthesized from N-isopropylacrylamide (NIPAAm) copolymers and methacrylate-poly(ethylene glycol)-perfluorooctane (MAPEGPFC) 55, which demonstrated significantly higher oxygen partial pressure when transitioning from 21% O₂ to 1% O₂ environments. When bone marrow-derived MSCs (BM-MSCs) were encapsulated in these hydrogels and cultured under 1% O₂, the cells survived and proliferated over a 14-day period, with markedly improved outcomes compared to hydrogels with lower oxygen retention capacity.

To ensure continuous oxygen supply, peroxide-based systems have emerged as a promising solution. These systems primarily generate oxygen through the decomposition of peroxides. Encapsulating hydrogen peroxide (H₂O₂) within PLGA/catalase microspheres facilitates on-demand oxygen release while mitigating ROS toxicity 57. Zhong *et al*. developed H₂O₂-releasing oxygen-generating nanoparticles that promote the recruitment of endothelial and skeletal muscle cells, enhance cell survival under ischemic conditions prior to neovascularization, and restore morphogenic function in high-glucose ischemic environments (**Figure 2B**) 15. Despite these benefits, precise modulation of H₂O₂ release kinetics remains a technical challenge. Among solid peroxides, calcium peroxide (CaO₂) is particularly promising due to its high oxygen yield (0.0069 mol O₂/g) and sustained release profile 58. Newland *et al*. introduced polyethylene glycol diacrylate (PEGDA)/CaO₂-based oxygen-producing microspheres capable of elevating the dissolved oxygen content of culture media for 16-20 hours (**Figure 2C**) 59. These spheres effectively preserved the viability of SH-SY5Y cells and MSCs under oxygen- and glucose-deprived conditions, indicating that oxygen-generating biomaterials can improve post-transplantation cell survival. Furthermore, when integrated into PLGA scaffolds, CaO₂-based systems enabled continuous oxygen release for over 10 days 60, with further extensions up to 7 weeks achieved through hydrophobic surface modifications 61 and adjustments to scaffold thickness 62. To enhance the controllability of release kinetics, Fu *et al*. developed an ultrasound (US)-activated oxygen-generating nanosystem. This platform consisted of CaO₂ encapsulated within mesoporous silica nanoparticles, combined with thermosensitive materials such as heneicosane and polyethylene glycol. Mild hyperthermia induced by targeted US irradiation initiated the phase transition of heneicosane, facilitating US-responsive water diffusion and oxygen release 63. This intelligent, stimulus-responsive nanosystem serves as a reference model for future development of controllable, on-demand oxygen release technologies.

Despite the promise of oxygen delivery strategies for SC transplantation, substantial technical and biological challenges remain. One major limitation is the mismatch between the oxygen release kinetics of current systems and the dynamic oxygen demands of transplanted tissues. Most conventional delivery systems—such as CaO₂ or H₂O₂-based platforms—exhibit binary, “all-or-nothing” release behavior, which fails to recapitulate physiological oxygen gradients and may induce cyclic hypoxia-reoxygenation injury. Furthermore, alkaline byproducts produced from peroxide decomposition can disrupt local pH homeostasis 64, while residual H₂O₂, if not adequately degraded due to insufficient catalase, may induce oxidative damage 57. To address these limitations, next-generation oxygen delivery systems are being developed to incorporate microenvironment-responsive feedback mechanisms. These advanced systems facilitate precisely regulated oxygen release in response to local pathophysiological signals, including oxygen tension, ROS levels, enzyme activity, and temperature fluctuations.

#### Glucose supplement

While hypoxia has long been recognized as a major contributor to the death of transplanted SCs, emerging evidence suggests that glucose deprivation is an equally critical limiting factor. Under hypoxic conditions, where oxidative phosphorylation is severely compromised, anaerobic glycolysis becomes the primary pathway for ATP generation, rendering glucose essential for cell survival 65. Multiple studies have demonstrated that various types of SCs, including human BM-MSCs 65, rat BM-MSCs, and sheep BM-MSCs 66, can adapt to and survive in near-anoxic environments (≤ 0.5% O₂), provided that glucose is adequately available. Moya *et al.* provided compelling evidence that human MSCs rely almost exclusively on glucose-driven anaerobic glycolysis for ATP production, showing minimal capacity to metabolize alternative energy substrates such as glutamine, serine, or pyruvate 65. Their work revealed that ensuring sufficient glucose supply significantly improves cell survival, reducing 14-day* in vitro* mortality of hMSCs from 93% to 40%. Beyond its fundamental role in energy metabolism, glucose serves as a vital signaling molecule that regulates critical cellular processes through pathways such as Akt/mTOR 67. This dual function underscores the importance of maintaining effective glucose delivery not only for sustaining cell viability but also for preserving the therapeutic functionality of transplanted cells during the critical pre-vascularization phase post-transplantation.

**Figure 3:**
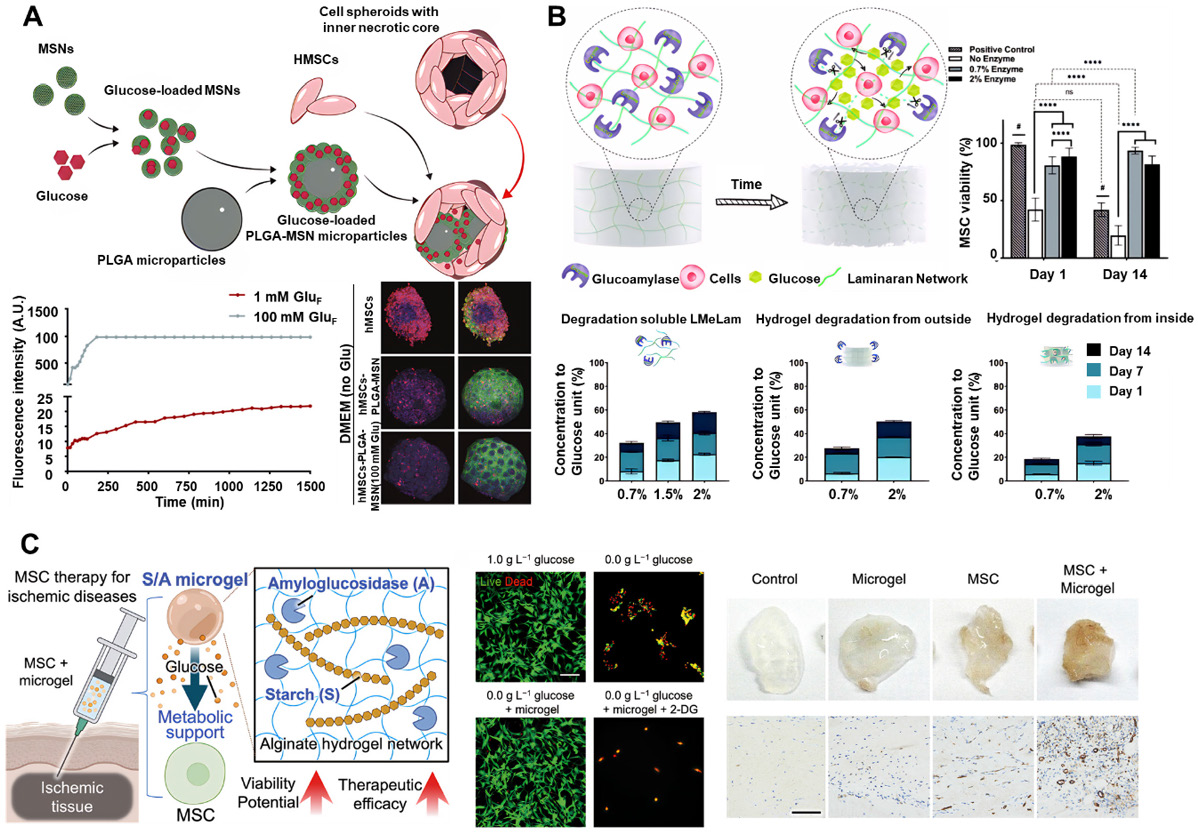
Representative examples of glucose delivery and release systems developed to support SC survival. (A) Schematic illustration of functionalized microparticles designed for glucose delivery in 3D cell assemblies. The accompanying graph depicts the glucose release profile from PLGA-MSNs and its effect on cell death. Adapted with permission from 68, Copyright 2024 American Chemical Society. (B) Schematic depiction of self-feeding hydrogels. The accompanying graphs present the enzymatic degradation profiles of various forms of laminaran and the proliferation of hMSCs encapsulated in laminaran hydrogels over a two-week period. Adapted with permission from 69, Copyright 2023 Nature Portfolio. (C) Schematic illustration of the potential application of glucose-releasing alginate microgels encapsulating starch and AMG. Adapted with permission from 72, Copyright 2024 Wiley-VCH GmbH.

Effective glucose delivery to transplanted cells encounters three primary challenges. First, the physicochemical properties of glucose, such as low molecular weight, neutral charge, and high aqueous solubility, make stable integration into scaffold matrices difficult. Second, the biological activity of glucose depends on preserving its native structure, thereby limiting options for chemical modification. Third, localized accumulation of glucose may lead to hyperosmotic stress, which can damage cellular membranes and induce lysis. In response to these challenges, Fois *et al*. developed nanofunctionalized microparticles to prevent necrotic core formation in SC spheroids 68. Glucose-loaded mesoporous silica nanoparticles (MSNs) were coated onto the surface of poly(lactic-co-glycolic acid) (PLGA) microparticles, resulting in the formation of cell-nanofunctionalized microparticle spheroids (**Figure 3A**). Sustained local glucose release within hMSC spheroids significantly enhanced cell viability under short-term hypoxic culture conditions.

Nevertheless, to achieve durable and stable glucose release, enzymatic glucose-release platforms have become a focal point of research in recent years. Denoeud *et al*. proposed a composite hydrogel consisting of fibrin, starch, and amyloglucosidase (AMG), enabling sustained glucose release *via* AMG-mediated starch hydrolysis (**Figure 3B**) 69. hMSCs encapsulated within these hydrogels, when exposed to near-anoxic conditions (0.1% pO₂) *in vitro*, showed improved viability and enhanced angio-inductive function for up to 14 days. Paquet *et al.* developed a fibrin-based hydrogel containing α-amylase, which enzymatically cleaves starch into glucose, supporting hMSC viability at levels 115 times greater than those observed with fibrin-only controls under near-hypoxic conditions after two weeks 70. Similarly, Zargarzadeh *et al*. utilized glucoamylase-immobilized laminaran hydrogels, which maintained > 60% viability in both A549 cells and MSCs throughout a 14-day glucose-free culture period 71. To extend the applicability of such nutritive platforms, Huang *et al*. developed an injectable glucose delivery system comprising starch and AMG encapsulated within alginate microgels (**Figure 3C**). These starch/AMG (S/A) microgels enabled sustained glucose release for up to 7 days *via* enzymatic hydrolysis, supporting MSC survival and function under ischemic conditions. *In vitro* studies under combined oxygen and glucose deprivation demonstrated that S/A microgels not only preserved cell viability and intracellular energy levels but also enhanced the pro-angiogenic and immunomodulatory functions of MSCs 72.

These biocompatible, self-nourishing platforms represent a significant advancement in cell transplantation technology. By enabling controlled and sustained glucose release, they transform passive cell delivery scaffolds into active metabolic support systems. Functioning as closed culture systems, these methods offer considerable promise for applications requiring extended cell viability before vascularization is established. Furthermore, combining glucose-delivery systems with oxygen-supplying strategies may greatly improve the efficacy of SC transplantation therapies. Future research should prioritize optimization of release kinetics, enhancement of scaffold integration, and development of clinically translatable formulations to maximize therapeutic outcomes.

### Vascularization

#### Angiogenesis

While the temporary supply of external nutrients can extend the survival of transplanted SCs, the establishment of a functional vascular system is essential for sustaining SC viability *in vivo*. This vascular system enables efficient exchange of nutrients and metabolic waste, which is crucial for long-term cellular function and homeostasis 73. Physiologically, blood vessel formation is governed by a series of tightly regulated molecular and cellular events 74, 75. Angiogenic activation is typically initiated by hypoxia-induced signaling, inflammatory signals, or extracellular matrix (ECM) degradation, leading to the binding of VEGF to its receptor, VEGFR2, on the surface of ECs 76, 77. This interaction activates multiple downstream pathways, including PI3K/Akt and MAPK/ERK, which promote EC proliferation and migration. Subsequently, the activated and differentiated ECs form neovascular sprouts that extend toward gradients of angiogenic factors, ultimately fusing with adjacent vascular sprouts to establish looped vascular structures. Neovascular maturation is then achieved through signaling mediated by platelet-derived growth factor (PDGF) and its receptor, PDGFRβ, which facilitates the recruitment of pericytes to encapsulate the newly formed vessels. Concurrently, ECs secrete components such as type IV collagen and laminin, contributing to the formation of a basement membrane and further reinforcing vascular integrity 78. The induction of artificial vascularization at this stage is therefore a key strategy for ensuring the long-term survival, engraftment, and functional integration of transplanted SCs at the target site.

Among the various angiogenic regulators, VEGF is widely recognized as the primary mediator of neovascularization due to its potent effects on EC proliferation, vascular permeability enhancement, and directional guidance of vessel formation 79-81. Consequently, contemporary research efforts have been directed toward optimizing VEGF delivery strategies to modulate SC fate and promote vascular development. For example, Park *et al*. developed a delivery system that co-encapsulates an angiogenesis-related peptide, apelin, with plasmid DNA to upregulate VEGF expression 82. Transfection using VEGF-coated, apelin-loaded PLGA nanoparticles induced differentiation of hMSCs into ECs and stimulated vascular formation both *in vitro* and *in vivo*. In another approach, Ullah *et al*. proposed that decellularized ECM retains structural and biochemical properties suitable for VEGF delivery 83. Supplementing VEGF with decellularized ECM enhanced the differentiation of human induced pluripotent stem cells (hiPSCs) into ECs, thereby facilitating the formation of functional vascular niches by promoting selective cell adhesion and survival. Furthermore, Lee *et al*. engineered a chitosan-based hydrogel embedded with VEGF-releasing microtubes 84. This cellular construct exhibited high cell viability and minimal cytotoxicity *in vitro*. Upon implantation into a mouse hindlimb ischemia model, it led to significant cell retention and neovascularization through both vasculogenesis and angiogenesis, ultimately contributing to the restoration of blood flow in ischemic tissue.

#### Construction of blood vessels

The application of 3D bioprinting technology in SC vascularization markedly enhances the efficiency of constructing functional vascular networks by enabling precise spatial control over the distribution of cells, biomaterials, and growth factors 85, 86. A key advantage of 3D bioprinting is its ability to fabricate vascular channels with a resolution of 10-50 μm, thereby allowing replication of the hierarchical structure found in natural capillary networks 87. For instance, Gou *et al*. developed a microfiber-templated porogel (μFTP) bioink for 3D printing of tubular interfaces and microvascular structures 88. This 3D tubular architecture exhibited up to 55% greater porosity than conventional block bioinks. The μFTP bioink effectively supported EC proliferation and spreading within the matrix and around sacrificial fibers. Scaffolds bioprinted with μFTP bioink significantly promoted inward growth of blood vessels and native tissues *in vivo*, demonstrating the feasibility of constructing tubular biointerfaces using customizable modular components. Additionally, 3D bioprinting enables synergistic multi-cell printing 89. For example, co-printing techniques involving ECs and MSCs have been employed to form the inner wall of blood vessels, facilitating lumen formation and vascular integrity 90-92. Cheng *et al*. implemented a one-step 3D bioprinting approach to create a physiologically relevant metastatic model incorporating tumor tissue, hollow vascular structures, and bone components 93. By utilizing a gelatin methacryloyl-based photo-crosslinkable bioink containing three distinct cell populations—MDA-MB-231 breast cancer cells, HUVECs, and osteoblasts (hOBs)—the researchers successfully recapitulated key features of the metastatic niche, including functional vasculature, vascularized tumor masses, and vascularized bone tissue. Moreover, 3D bioprinting allows for the mimicry of SC ecological niches. Paracrine signals secreted by blood vessels, including VEGF and stromal-derived factor 1 (SDF-1), can be integrated into SC-hydrogel complexes printed around blood vessels to sustain SC viability and function 94, 95. Consequently, the integration of 3D bioprinting for direct construction of vascularized structures with active material exchange capacity not only facilitates continuous nutrient delivery and prolongs SC survival while maintaining therapeutic efficacy but also lays the groundwork for future regeneration of large-scale organs, such as the heart and liver 96.

Despite these advancements, several critical challenges persist in the field of vascular engineering. The scientific community continues to grapple with issues such as uncontrolled vessel proliferation, incomplete vascular maturation, and progressive loss of perfusion efficiency over time. Addressing these limitations will require focused efforts to refine growth factor delivery methods, develop advanced biomimetic strategies that more accurately replicate physiological angiogenesis, and explore innovative approaches for modulating the immune microenvironment surrounding newly formed vasculature.

### Relieving oxidative stress damage

Reactive oxygen species (ROS) are natural byproducts of cellular oxygen metabolism that play crucial, yet dual, roles in SC physiology. These chemically reactive molecules, which include superoxide anion (O₂^•⁻^), H₂O₂, singlet oxygen (¹O₂), and hydroxyl radical (•OH), are generated through the sequential reduction of molecular oxygen 97, 98. Under physiological conditions, redox homeostasis is maintained through a network of enzymatic antioxidant systems. Specifically, superoxide dismutase (SOD) catalyzes the conversion of O₂^•⁻^ into H₂O₂, which is subsequently decomposed into water by catalase (CAT), peroxidase (POD), and glutathione peroxidase (GPX) 99. These antioxidant enzymes also modulate redox-sensitive signaling pathways that involve cytokines, growth factors, protein kinases and phosphatases, and nuclear transcription factors 100, 101. Nevertheless, when ROS production exceeds the cellular antioxidant defense capacity, oxidative stress ensues, resulting in damage to vital cellular components. In the context of SC transplantation, abnormal metabolic activity at the lesion site often leads to ROS overproduction. This is further exacerbated by inflammatory and immune responses that generate excessive ROS, which can infiltrate and compromise the viability of transplanted SCs. This aggressive influx of ROS, along with associated oxidative damage, is commonly observed in pathological conditions such as atherosclerosis, ischemic tissue necrosis, pulmonary and hepatic fibrosis, and neurodegenerative diseases 102-104. To mitigate the oxidative stress associated with these conditions, various antioxidant-based strategies have been developed to protect transplanted SCs from ROS-induced injury. The following section provides an overview of recent advances in antioxidant compound development and their efficacy in enhancing SC resistance to oxidative stress in transplantation settings.

#### Antioxidant enzymes

Enhancing the activity of endogenous antioxidant enzymes through gene editing represents an effective strategy for mitigating oxidative stress in transplanted SCs. For instance, Baldari *et al*. demonstrated that adipose-derived stromal and vascular cells engineered to overexpress SOD2 exhibited significantly enhanced survival under hypoxic conditions, highlighting the protective role of SOD2^105^. In a more comprehensive approach, Zhu *et al.* engineered bone marrow-derived MSCs (bMSCs) to co-express both SOD1 and GPX, establishing a synergistic antioxidant defense system 106. These genetically modified bMSCs showed improved survival following transplantation and contributed to enhanced circulatory and functional recovery in a diabetic critical limb ischemia model. However, gene editing technologies remain primarily limited to preclinical research, as the accumulation of genetic modifications may pose risks and potentially impair SC function.

Alternatively, recent advances in delivery platforms have enabled the direct intracellular transport of antioxidant enzymes, offering a promising strategy to alleviate oxidative stress 107-109. For instance, Chen *et al*. developed a nanoparticle-based SOD delivery system (FMSN-TAT-SOD), which consists of mesoporous silica nanoparticles combined with a cell-penetrating peptide 109. This system significantly increased intracellular SOD levels in HeLa cells, enhancing resistance to free radicals and superoxide anions induced by lipopolysaccharide (LPS) and paraquat. In our previous work, SOD-conjugated gold nanoparticles (SOD@Au NS) were engineered as ROS scavengers for MSCs in the treatment of idiopathic pulmonary fibrosis (IPF) 22. In this approach, SOD was immobilized on the surface of gold nanoparticles (AuNPs) and encapsulated within polyphosphazene nanospheres to improve cell membrane permeability and chemical stability. In addition to endogenous antioxidant enzymes, exogenous proteins such as Wnt3a—which modulates oxidative stress *via* the Wnt/β-catenin signaling pathway—have also been utilized. Qi *et al*. demonstrated the use of cell-penetrating peptide-conjugated porous silicon nanoparticles (TPSi NPs) for efficient Wnt3a loading and sustained release, which enhanced the antioxidative capacity of MSCs 107. This strategy holds significant promise for improving outcomes in SC therapy for myocardial infarction by protecting transplanted cells from ROS-induced damage.

#### Small molecule antioxidants

Unlike biologically active macromolecules, small-molecule antioxidants typically exhibit superior physicochemical stability. Compounds such as vitamin C (ascorbic acid), vitamin E (tocopherol), vitamin A (β-carotene), selenium, glutathione (GSH), and N-acetylcysteine (NAC) have shown efficacy in counteracting oxidative damage 110-112. These antioxidants play a crucial role in protecting SCs from oxidative stress and enhancing post-transplantation survival. N-acetylcysteine, for example, has been shown to safeguard MSCs against oxidative stress and metabolic dysfunction by mitigating DNA damage and apoptosis through the Nrf2/Sirt3 signaling pathway 112. In one study, Zhu *et al*. administered NAC *via* drinking water in a diabetic mouse model of critical limb ischemia. This intervention significantly reduced tissue ROS levels and improved the survival rate of bMSCs by nearly five-fold, increasing from 7.63% to 35.66% compared to untreated controls 106. However, a major challenge in the clinical application of small-molecule antioxidants lies in achieving effective concentrations at the transplantation site following systemic administration. This is primarily due to low bioavailability, which results from high water solubility and poor absorption. Therefore, the development of optimized delivery methods remains essential to maximize the antioxidative potential of these compounds in transplanted SCs.

In addition to direct delivery, pretreating SCs with antioxidants prior to transplantation has emerged as an effective strategy for enhancing resistance to ROS-mediated damage. Deferoxamine (DEF), a clinically approved iron chelator, has been shown to improve MSC viability by enhancing antioxidant defenses and upregulating neurotrophic and anti-inflammatory factors 113. For example, Soleimani Asl *et al*. reported that DEF-preconditioned adipose-derived MSCs exhibited increased antioxidant activity, proliferation, and overall viability. In an Alzheimer's disease model, DEF-treated AMSCs significantly improved cognitive function compared to untreated cells 114. While *in vitro* preconditioning offers advantages such as procedural simplicity and enhanced controllability, several challenges persist. These include achieving sufficient intracellular drug accumulation and establishing reliable stimulus-responsive release mechanisms to sustain antioxidant protection after transplantation.

#### Nanozymes

Nanozymes are a distinct class of engineered nanomaterials that exhibit catalytic properties similar to those of natural enzymes 115. These synthetic catalysts offer significant advantages over conventional protein-based enzymes, including a broader catalytic spectrum, sustained activity across diverse temperature and pH ranges 116, and enhanced cost-effectiveness in terms of production and storage stability 117. Recent studies have identified a wide range of nanozyme materials, including metal and metal oxide nanoparticles (e.g., Au, Ag, Pt, CuxO, CeO₂, V₂O₅, Fe₃O₄, and MoO₃), carbon-based nanostructures (such as metal-doped carbon dots and graphene quantum dots), and metal-organic framework (MOF) nanoparticles. While existing reviews have comprehensively summarized nanozyme classifications, mechanisms of action, and biomedical applications 99, 118, 119, the present discussion focuses specifically on the emerging role of nanozymes in enhancing SC therapy through ROS scavenging.

Cerium oxide nanoparticles are particularly notable for their exceptional antioxidant properties, which arise from reversible redox cycling between the Ce⁴⁺ and Ce³⁺ oxidation states 118, 120-123. Periodontitis, a chronic inflammatory disease, significantly impairs the function of periodontal ligament stem cells (PDLSCs) due to excessive ROS accumulation, which undermines their regenerative capacity and hinders periodontal tissue repair. To address this issue, Ren *et al*. developed PEGylated mesoporous silica nanoparticles loaded with cerium oxide (MSN@Ce@PEG) 124. These nanoparticles effectively protected PDLSCs from oxidative damage, as evidenced by experimental data indicating a reduction in H₂O₂-induced apoptosis from 65.4% in untreated controls to 5.16% following treatment. *In vivo* studies conducted on rat models of periodontitis further confirmed the anti-inflammatory properties of these nanoparticles and their ability to enhance the periodontal microenvironment, ultimately improving PDLSC survival and function. In the context of neurodegenerative diseases, neural stem cell (NSC) transplantation presents promising therapeutic prospects for addressing cognitive impairment. To support NSC viability, Yu *et al*. developed cerium-based nanoparticles encapsulated within MOFs 125, which revealed efficient ROS scavenging and neuroprotective effects. Quantitative analyses demonstrated significantly improved neuronal survival rates and enhanced neurite outgrowth, underscoring the therapeutic potential of this antioxidant strategy in promoting neural regeneration.

Manganese dioxide (MnO₂)-based nanozymes have shown considerable potential in protecting SCs from oxidative damage 126, 127. For instance, Peng *et al*. developed an injectable hydrogel microsphere system (LMGDNPs) by incorporating MnO₂ nanozymes into glucose-enriched decellularized nucleus pulposus hydrogel microspheres 128. MSCs cultured on LMGDNPs exhibited improved cell viability and ECM synthesis, thereby promoting intervertebral disc regeneration. In a related approach, Chen *et al*. dispersed MnO₂ nanoparticles into a gelatin/κ-carrageenan hydrogel to enhance its ROS scavenging ability. The resulting hydrogel mitigated oxidative stress, improved SC viability, and boosted paracrine function, ultimately promoting angiogenesis in a skin flap repair model 129. Similarly, Yang *et al*. fabricated MnO₂-containing granular hydrogel matrices capable of supporting MSC adhesion, preserving stemness, and modulating the ROS microenvironment by catalyzing the conversion of H₂O₂ into oxygen. This approach promoted MSC viability and osteogenic differentiation 130. In a two-week diabetic rat model, the granular hydrogel also demonstrated enhanced *in vivo* cell retention and anti-inflammatory immunomodulation effects, highlighting its therapeutic potential for SC transplantation and immune regulation in diabetic conditions. In the context of spinal cord injury (SCI), Li *et al.* introduced a nanoparticle-dotted hydrogel by incorporating MnO₂ nanoparticles into a hyaluronic acid-based matrix 131. This system effectively alleviated oxidative stress at the lesion site, improved MSC viability, and promoted the regeneration of central nervous spinal cord tissue.

Copper-based nanoparticles represent another promising class of nanozymes 132, exhibiting broad and potent catalytic capabilities. Liu *et al.* demonstrated that Cu₅.₄O nanoparticles could degrade up to 80% of three major ROS—H₂O₂, O₂^•⁻^, and •OH—at a concentration of 150 ng/mL 133. In the context of SC therapy, Chen *et al.* fabricated a continuous nanozyme coating by sequentially depositing copper phosphate nanosheets (Cu NS) and the phenolic ligand epigallocatechin gallate (EGCG) onto titanium (Ti) implants 134. These metal-phenolic nanozyme bio-interfaces exhibited robust free radical scavenging activity and facilitated SC adhesion, migration, and osteogenic differentiation, thus enhancing implant osseointegration in diabetic rat models. In our previous work, ROS-scavenging nanocomposites (RSNPs) were developed by encapsulating Cu_x_O nanoparticles and AuNPs to serve both as antioxidance agents and computed tomography (CT) imaging tracers 135. Upon internalization by MSCs, RSNPs released Cu_x_O nanoparticles in response to oxidative stress, thereby protecting the cells and improving therapeutic efficacy in IPF models by enhancing SC survival.

In addition to metal-based nanozymes, non-metal nanozymes—particularly those derived from carbon-based materials—have also demonstrated significant success in the field of SC therapy 136, 137. Among these, reduced graphene oxide (rGO) has been shown to mitigate excessive ROS through its ability to scavenge free radicals via electron transfer mechanisms 138, 139. For instance, Choe *et al.* encapsulated MSCs in rGO/alginate composite microgels using an electrospraying technique 140. The encapsulated MSCs exhibited increased viability under oxidative stress conditions induced by H_2_O_2_. Furthermore, when cardiomyocytes (CMs) were co-cultured with the encapsulated MSCs in a transwell system and exposed to H_2_O_2_, the CMs showed enhanced survival and greater degrees of cardiac maturation compared to those cultured in monolayers. In a related study, Zhou *et al.* confirmed that rGO-embedded antioxidant hydrogels possess significant ROS-scavenging capacity and promote both the osteogenic differentiation of bMSCs and angiogenesis in HUVECs. These hydrogels neutralized local ROS at the site of skull defects and stimulated the ZEB1/Notch1 signaling pathway, thereby facilitating coordinated osteogenesis and angiogenesis 137.

Nanozymes hold significant potential for improving the efficacy of SC therapy by mimicking the activity of natural antioxidant enzymes and effectively neutralizing deleterious ROS. However, several critical challenges must be addressed to fully realize their therapeutic potential. One major challenge lies in the precise modulation of ROS levels. It is imperative that nanozymes are meticulously engineered to selectively eliminate pathological concentrations of ROS while preserving basal ROS levels necessary for redox signaling, which governs a variety of cellular processes. To achieve this, the development of sophisticated ROS-recognition systems capable of enabling accurate and controlled ROS elimination is urgently needed 135, 141, 142. In addition, long-term biocompatibility remains a significant concern, particularly for metal-based nanozyme formulations. The accumulation of exogenous nanomaterials may trigger chronic inflammatory responses, unintended immune activation, or organ-specific toxicity. Consequently, comprehensive preclinical assessments—including extensive toxicological profiling and thorough pharmacokinetic analyses—are imperative before clinical translation can be considered 143, 144. Moreover, the therapeutic challenges in SC transplantation extend beyond ROS-related injury 6. Integrating multifunctional capabilities into nanozymes represents a promising direction for future research. For example, combining ROS-scavenging activity with functionalities such as SC tracking or controlled drug delivery could maximize the benefits of nanoparticles, offering a platform for more effective and versatile regenerative strategies.

### Stem cell spheroids

SCs isolated from human tissues are typically cultured in two-dimensional (2D) monolayer systems to produce sufficient quantities for biological research and therapeutic applications 145-147. However, a growing body of evidence suggests that prolonged culture under 2D conditions results in the gradual loss of stemness characteristics. This decline is primarily attributed to inadequate interaction with the ECM and the absence of physiological relevance, resulting in abnormal cellular metabolism and altered protein expression profiles 148, 149. These suboptimal culture conditions contribute to accelerated cellular aging, diminished paracrine signaling, and reduced post-transplantation survival, ultimately compromising therapeutic efficacy 150, 151. To overcome these limitations, 3D cell culture systems have gained significant attention for their ability to mimic the *in vivo* microenvironment more accurately, including dynamic cell-cell and cell-ECM interactions 152. 3D cell formats—such as aggregates, spheroids, and organoids—exhibit unique properties that influence specific cellular functions and growth processes, including embryogenesis, morphogenesis, and organogenesis. These advantages stem from the preservation of natural cytoskeletal organization, maintenance of apical-basal polarity, provision of appropriate matrix stiffness, and the establishment of physiological cytokine gradients 153, 154. Spheroids derived from 3D culture demonstrate enhanced ECM production, which plays a protective role by mitigating inflammatory damage, reducing macrophage-mediated clearance, and improving post-transplantation migration and survival 155, 156. For instance, sphere-forming cultures maintained under hypoxic conditions upregulate SDF-1α and HIF-1α expression, thereby increasing stress resistance, survival, homing, and angiogenic capacity of MSCs *in vivo* 157. Furthermore, MSC spheroids display increased expression of SOD2, which reduces oxidative stress and apoptosis, while activating the PI3K/AKT/NRF2 and ERK/NRF2 signaling pathways to promote cell survival 158. Thus, the development and utilization of SC spheroids and organoids represent promising alternatives for advancing regenerative medicine and enhancing the efficacy of SC-based therapies.

The formation of spheroids arises from the self-assembly behavior of single cells in suspension, facilitated by engineered platforms that provide environments conducive to cell aggregation and self-organization of the resulting multicellular structures 159-161. Based on the engineering tools employed, 3D cell culture systems can be classified into scaffold-based systems (*e.g.*, hydrogel matrices, well plates, and microfluidic chips) and scaffold-free systems (*e.g.*, hanging drop, rotation methods, and magnetic force) 152. In the scaffold-free category, He *et al*. prepared MSC spheroids using the hanging drop method 159. The enhanced formation of ECM receptor interactions, gap junctions, and tight junctions within the spheroids activated neuroactive ligand-receptor interactions, which in turn triggered downstream PI3K-Akt signaling, resulting in superior anti-inflammatory and neurogenic effects. However, the absence of ECM-like materials in scaffold-free systems raises concerns regarding cell-cell adhesion and spheroid structural integrity. To address this, advances in biomedical engineering have introduced a variety of biological and synthetic scaffold materials with tunable porosity, permeability, surface chemistry, and stiffness to better replicate tissue-specific microenvironments and promote cell growth and migration. Inspired by lotus seedpod-structured hydrogels with temperature-responsive properties, Kim *et al*. developed a novel strategy for the culture and delivery of hADMSC spheroids using a microwell platform 162. Spheroids cultured within these microwells demonstrated a high transfer efficiency of 93.78 ± 2.30% to target substrates. In a full-thickness murine skin wound model, a significant increase in SMA-positive vessel formation was observed by day 21. In addition, Rossen *et al*. introduced a sacrificial scaffold that allows ECs and MSCs to form self-organized clusters. Their results indicated that this facilitated the self-assembly of ECs and MSCs into prevascular units, which rapidly developed into functional, perfusing vasculature upon injection into mic 163. This microplate array-based approach leverages the dynamic properties of hydrogels and offers scalability and reproducibility in producing self-organized cell clusters. Beyond single-cell-type spheroids, multicellular spheroid systems have further advanced SC therapy by more effectively mimicking native cellular interactions. Sung *et al.* created self-assembled 3D spheroids composed of umbilical cord MSCs and HUVECs 164, 165. These constructs rapidly formed primitive vascular networks that matured into functional vasculature, supported by MSC-derived trophic and proangiogenic factors 164, 166. When co-transplanted with islet cells, the spheroids yielded remarkable therapeutic benefits, including a 78% improvement in hypoxia resistance under 1% O₂, a 2.3-fold increase in insulin secretion, and a 62% enhancement in *in vivo* survival rates 166.

While 3D SC spheroids offer substantial advantages over traditional 2D cultures, several critical challenges must be addressed before their widespread clinical adoption. First, the inherent heterogeneity of the cellular microenvironment within spheroids presents a significant limitation. As cells with varying metabolic states aggregate into 3D structures, uneven oxygen and nutrient gradients emerge, coupled with inefficient waste removal. These suboptimal conditions result in spatial variations in gene expression patterns and metabolic activity, ultimately compromising the quality and functional consistency of the resulting spheroids 167. Second, the processing of 3D spheroids introduces substantial risks of cellular damage. Larger spheroids require prolonged enzymatic dissociation, which subjects cells to increased mechanical stress and potential damage. Such processing-induced injury is particularly concerning, as apoptotic or compromised cells may elicit undesirable immune responses following transplantation, potentially undermining therapeutic outcomes 168, 169. Third, standardization remains a formidable obstacle in spheroid production. Current fabrication methods exhibit considerable batch-to-batch variability in both spheroid size and quality, posing challenges for research reproducibility, limiting the reliability of preclinical validation in animal models, and ultimately hindering the clinical translation of MSC-based therapies 170, 171. These technical challenges collectively underscore the need for continued innovation in 3D culture technologies. Systematic resolution of these issues through advanced bioengineering approaches and rigorous quality control measures will be essential to establish a robust foundation for clinical applications of 3D SC spheroids. Only through comprehensive optimization can the full therapeutic potential of this promising technology be effectively realized in clinical settings.

## Summary and Prospects

The therapeutic efficacy of transplanted SCs is closely linked to their *in vivo* survival rate, with higher survival rates typically correlating with more consistent and durable therapeutic outcomes. However, SC survival is influenced by a range of factors, including intrinsic cellular states (*e.g.*, metabolic disorders and substrate deficiencies), microenvironmental conditions at the transplantation sites (*e.g.*, immune responses, oxidative stress, and suboptimal physicochemical microenvironments), as well as the biomechanical properties of the host matrix. In response to these challenges, researchers have developed various strategies to enhance SC viability, including the supplementation of oxygen and glucose, promotion of neovascularization, scavenging of excessive ROS, and the use of SC spheroids and hydrogel scaffolds to better mimic physiological microenvironments. These advances have collectively provided valuable research tools and technical support for preclinical SC research and clinical translation of SC-based therapies.

Nevertheless, several key issues remain that warrant systematic investigation to guide future developments. (1) Biocompatibility remains a fundamental prerequisite for clinical translation. A comprehensive assessment should include not only the inherent toxicity of biomaterials but also the safety profile of their degradation byproducts. For instance, although sodium peroxide and calcium peroxide exhibit minimal direct effects on SC growth, their byproducts—sodium hydroxide and calcium hydroxide—may disrupt local pH homeostasis 62. Similarly, metal-based nanozymes such as MnO₂ and CuₓO nanoparticles demonstrate promising application potential due to their low cytotoxicity. However, as exogenous substances, their *in vivo* metabolism and pharmacokinetics must be thoroughly characterized before clinical application. (2) Spatiotemporal regulation of the transplantation microenvironment is essential to meet the dynamic needs of SC growth and differentiation. For example, while ROS at low concentrations serve critical physiological signaling functions, excessive ROS can lead to oxidative stress and cellular damage. Therefore, the use of antioxidant materials alone may be insufficient to maintain normal cellular function. The development of intelligent delivery systems—featuring stimuli-responsive activation, microenvironment-sensitive behavior, and tunable therapeutic release kinetics—is essential for achieving precise, on-demand modulation of therapeutic agents. (3) Integration of multifunctional biomaterials is necessary to address the multifaceted challenges encountered in SC therapy systematically. For example, a multicomponent system using hydrogel scaffolds to support the formation of HUVEC-lined vascular structures, while concurrently incorporating antioxidant molecules, could synergistically address key factors limiting graft survival, including biomechanical stress, insufficient angiogenesis, and oxidative damage. In addition, persistent obstacles such as immune rejection and the inability to achieve non-invasive, real-time tracking of transplanted cells continue to hinder the clinical advancement of cell therapies. The incorporation of multifunctional capabilities—such as immunomodulation, imaging contrast enhancement, and survival-promoting agents—within a single, integrated system represents a promising direction. Such holistic strategies not only enhance SC survival post-transplantation but also expand the applicability and clinical utility of SC-based therapeutic interventions 172.

Overall, SC research is not solely a biological endeavor but inherently intersects with tissue engineering, nanotechnology, drug delivery, and molecular imaging. These interdisciplinary connections present both substantial opportunities and complex challenges. Realizing the full therapeutic potential of SC-based therapies will require ongoing collaboration and communication among researchers from diverse fields, including chemistry, engineering, biology, and clinical medicine. Through such integrative efforts, rapid development and breakthroughs in clinical applications are anticipated, transforming the understanding of fundamental physiological processes and delivering meaningful benefits to human health in the near future.
